# Creative decision making and visual search behavior in skilled soccer players

**DOI:** 10.1371/journal.pone.0199381

**Published:** 2018-07-10

**Authors:** André Roca, Paul R. Ford, Daniel Memmert

**Affiliations:** 1 Expert Performance and Skill Acquisition Research Cluster, School of Sport, Health and Applied Science, St Mary’s University, Twickenham, London, United Kingdom; 2 Institute of Training and Computer Science in Sports, German Sport University Cologne, Cologne, Germany; 3 University of Brighton, Brighton, United Kingdom; Universita Cattolica del Sacro Cuore, ITALY

## Abstract

The ability to produce creative solutions is a key part of expert performance. The aim of this study was to identify the visual search behaviors that underpin superior creative performance of skilled soccer players during simulated 11-a-side match play. Players (*N* = 44) were required to interact with a representative life-size video-based simulation of attacking situations whilst in possession of the ball. Clips were occluded at a key moment and they were required to play the ball in response to each situation presented. Moreover, they were required to name other additional actions they could execute for each situation. Creative performance on the task was measured using the three criteria of originality, flexibility, and fluency of decisions. Visual search behaviors were examined using a portable eye-movement registration system. Players were classified as most- (*n* = 11) or least-creative (*n* = 11) based on their performance on the representative task. The most-creative players produced more appropriate, original, flexible, and fluid decisions compared to least-creative players. The creativity-based differences in judgment were underpinned by differences in visual search strategy. Most-creative players employed a broader attentional focus including more fixations of shorter duration and towards more informative locations of the display compared with least-creative players. Moreover, most-creative players detected teammates in threatening positions earlier in the attacking play. Creative performance is underpinned by different underlying visual processes when compared to less-creative performance, which appears to be crucial in facilitating more creative solutions.

## Introduction

The ability to produce creative solutions is key to expert performance in sport. In soccer, for example, an attribute of high-performance players is the ability to be novel and surprising in their decision-making processes under time constraints, thereby allowing them to be more effective in unique performance situations and make it more difficult for opponents to predict what they do next. Creativity is defined as the ability of the performer to produce solutions that are both novel (i.e., original, rare) and appropriate (i.e., adequate, useful) across different situational contexts [1]. These creative behaviors are assumed to be more important as players reach higher levels of performance where athletes/teams become more homogenous regarding their physical and physiological characteristics [2]. Although creative decision making is a key component of expertise, little is still known about the underlying perceptual-cognitive processes that mediate creative performance in the sporting domain (for a review, see [3]).

An extensive number of research studies investigating perceptual-cognitive processes underpinning creativity have been conducted in the domain of general creative thinking (for extended overviews, see [4, 5]) or domains outside sport (e.g., traditional arts, sciences, business and technology; for a recent overview, see [6]). Recently, researchers in the field of sport have started to examine some of the perceptual processes that lead to the generation of creative actions in more continuous and highly-dynamic situations (for a review, see [7, 8]). These studies have largely focused on attentional processes associated with creativity in open-play sport settings using the inattentional blindness paradigm (e.g., see [9, 10]). This paradigm tests the prediction that when attention is diverted to another object, observers sometimes fail to perceive an unexpected object, even if it appears right in front of them. For example, Furley et al. [9] were able to show that adult basketball players’ tactical decision making declined if they had to perform an attention demanding task (i.e., name the position of their direct opponent at the end of the trial) which was intended to facilitate their tactical decision. Attention-directed instructions reduced attentional focus, leading to players missing important creative opportunities such as completely unmarked teammates. The authors concluded that a narrow breadth of attention limits the amount of stimuli and critical visual information that can be extracted and integrated, thereby reducing the potential of discovering unique and original solutions.

To date, there have been no attempts to effectively capture the visual search patterns that occur during superior creativity in sport performance contexts. Several researchers (e.g., [11–16]) have used eye-movement recording to examine the visual search behaviors employed by performers on convergent thinking tasks. For example, during soccer open-play situations, skilled players’ superior anticipation and decision-making performance was underpinned by visual search patterns involving more fixations of shorter duration and to a greater number of informative locations such as unmarked teammates, opponents, and ‘free’ space areas, when compared to lesser-skilled players (e.g., [12, 14, 15]). Further exploration of the visual-perceptual processes in sport-specific creativity is clearly warranted in order to identify the key visual cues used by performers to guide creative behavior (cf. [8]). Such knowledge will enhance our understanding of the perceptual processes that are associated with creativity in dynamic, invasion team sport situations, which in turn have implications for the design of training interventions to facilitate the development of more creative behaviors in these sports.

Several researchers interested in tactical creativity in sport have used sport-specific video tests of divergent thinking to capture the components of creative performance. Typically, participants watch sport-specific video clips of a few seconds duration, after which the last frame is frozen for up to a minute and players are asked to generate as many possible decisions as possible (e.g., see [17, 18]). Although this has been the methodological norm in research on sport creativity, players in continuous and dynamic open-play sports are normally required to select and execute tactical decisions in temporally constrained situations. Additionally, the lack of physical realism encountered in the video-based tasks used in these studies, where participants are required to watch and write down their solutions, might alter the natural role of the underlying perceptual-cognitive processes underpinning players’ creative behavior [19]. For example, in a recent study, Roca, Williams, and Ford [20] compared the cognitive processes of skilled soccer players when responding to video-based defensive soccer simulations under two different response modes that were either stationary or movement based. Participants in the movement condition engaged in a larger number of higher-order thought processes compared to stationary participants. The lower representativeness and fidelity of the non-movement response mode appears to have altered to some degree the normal performance and processing strategies of players. Research on tactical creativity is still at an early stage and further research is required to develop and validate new sport-specific creativity tasks, as well as to refine the theoretical framework for the understanding of creative behavior in sport [7].

The aim of this study was to examine creativity in the decision making and visual search behaviors of skilled soccer players during simulated 11-a-side match play. In contrast to previous research using video tasks, participants in this experiment were required to move and physically respond to representative life-size video-based simulations of soccer attacking situations that were occluded at a key moment. Creative performance on the task was used to categorize players into either the most- or least-creative groups and visual search behaviors recorded using eye-movement registration techniques. We expected, based on previous literature [8, 9], that creativity-based between-group differences in decision making would be underpinned by differences in visual search strategy. Specifically, we expected that the most-creative players would employ a search strategy involving more fixations of shorter duration and towards more informative locations of the display compared with least-creative players, indicating a broader attentional focus. Moreover, we hypothesized that due to the use of a wider breadth of attention, most-creative players would be able to perceive relevant cues (e.g., attacking teammates in a threatening/dangerous position) earlier on in the attacking play.

## Methods

### Participants

A total of 44 skilled, male outfield soccer players (*M* age = 20.8 years, *SD* = 2.2) participated. These players were recruited from a range of different professional and semi-professional soccer clubs in England. Participants had an average of 15.2 years (*SD* = 2.7) of playing experience and an average of 8.3 h (*SD* = 2.3) training or playing per week. Written informed consent was obtained from the participants prior to taking part in the study and all participants had a right to withdraw at any point. The experiment was conducted in accordance with the 1964 Declaration of Helsinki and approval was obtained from St Mary’s University Research Ethics Committee.

### Creativity task

Participants were presented with life-size video sequences of dynamic 11 versus 11 attacking situations that allowed for a variety of possible solutions for the player in possession of the ball at the time of video occlusion. A panel of three UEFA (Union of European Football Associations) qualified soccer coaches independently selected the scenes from a large battery of matches from the highest professional soccer league in Germany (i.e., Bundesliga). The final test film included 20 video clips for which the coaches had agreed upon offering a range of multiple options that may provoke creative tactical solutions [21]. The video clips lasted approximately 10 s each and were occluded at a key moment in the action (i.e., the participant in possession of the ball with a variety of tactical options available including different attacking passes, shot at goal, or dribbling forward).

### Apparatus and procedure

The soccer-specific creativity test film was projected onto a large white wall using a 3LCD video projector (Epson EB-X31, Tokyo, Japan) providing an image size of 2.5 m (h) x 3.4 m (w). Participants stood at a distance of approximately 3 m from the wall with a soccer ball (Mitre Cyclone indoor size 4 ball) placed directly in front of them. They were required to imagine themselves as the attacking player with the ball. In order to increase realism of the test setting, participants were required to play the ball in response to each situation as quickly as possible as the screen was occluded. Moreover, they were required to verbally confirm their initial response immediately after executing the action, which should be either to whom they were passing the ball or if they shot at goal or dribbled forward. Additionally, they had to define how they intended to pass the ball to the player or shoot the ball at goal (i.e., how decision). Following this, the last frame of the video clip was shown again for 45 s during which time the participants were required to generate as many adequate tactical solutions as possible for that situation (divergent thinking). The real ambient crowd noise of the stadium was played through multimedia stereo speakers (Logitech Z200, Lausanne, Switzerland) during the test film to provide a more natural and realistic impression of immersion.

A mobile eye-tracking system (Applied Science Laboratories, Bedford, MA, USA) was used to record participants’ visual search data. It is a video-based monocular system that measures eye point-of-gaze with respect to a head-mounted scene camera. It measures the relative position of the pupil and corneal reflection in relation to each other by using an infrared light source at a frame rate of 50 Hz and has a manufacturer-reported spatial accuracy of ± 0.5° and a precision of 0.1° of visual angle. Moreover, a scene image is provided by the head-mounted camera. Both sources are automatically linked and result in a computed point-of-gaze superimposed as a cursor onto the scene image. The data were analyzed frame-by-frame using Focus X2 video analysis software (Elite Sport Analysis, Fife, UK).

Prior to commencing the testing, the experimental protocol was explained and the eye-movement system fitted onto the participant’s head. The system was calibrated using a reference of six to nine non-linear calibration points on the visual display to ensure that the participants’ point-of-gaze was accurately recorded. Calibration of the system was checked prior to starting the familiarization trials, between familiarization and experimental trials, and periodically during testing. Participants were presented with 3 familiarization and 20 test trials and each individual test session was completed in approximately 45 min to 1 hr.

### Outcome data analysis

Creativity performance on the soccer-specific creativity test was measured using the three criteria of *originality*, *fluenc*y, and *flexibility* derived from key creativity research [5, 22]. These measures have been commonly used to evaluate athletes' tactical creative performance in numerous studies (for a review, see [7]). *Originality* referred to the production of responses that are rare or a-typical according to the norm. Three independent raters (UEFA qualified soccer coaches) judged the originality of the solutions given by participants for each scene using a Likert-type scale range between 1 (not original at all) to 5 (very original). A high degree of inter-rater reliability was found between coaches for originality with a reported intraclass correlation coefficient of 0.85. Since each scene was occluded at a key moment in the action, in order to obtain an immediate action response from participants (as opposed to freezing the last frame of the clip as for previous research in this area, e.g., [18]), an additional originality criterion was used for the participants’ first response. These ratings were used to compute two mean originality scores for each participant, one for the first or initial response and another for the responses given when the last frame was shown for 45 s afterwards (summed ratings for each response were divided by the total number of responses). *Fluency* was assessed by the number of appropriate tactical solutions produced by a participant per trial. *Flexibility* was measured via diversity of responses. All solutions given by the participants were categorized into different kinds of solution options (i.e., short pass, lofted pass, through ball, wall pass, back heel pass, outside of the foot pass, feinting, turn, crossing, dribbling, shot at goal). One point was given for each category selected by a participant and summed for the respective trial, before being divided by the total number of trials to obtain a flexibility score for every participant. The standard procedure in creativity research (cf. [18, 21]) was used in which each of the four components (originality of initial response, originality, fluency, flexibility) were analyzed independently followed by a z-transformation and averaging of all four values into one creative performance value.

The creativity scores (total, z-value) from the sport-specific creativity test were used as an objective method to create a rank order and differentiate the 44 skilled soccer players. A quartile-split approach was used to create two groups from this rank order. The top 25% (*n* = 11) ranked players were classified as ‘most creative’ whereas players ranked in the bottom 25% were classified as ‘least creative’. The participants ranked in the middle 12–33 were excluded from further analysis. A priori power analysis was conducted using G*power [23]. We based our calculations on the main effect sizes for total creativity score reported by Memmert [24] who used a similar sport-specific video-based creativity task to compare handball players of different age and skill levels. Results of the analysis reveals that we have an appropriate power with a total sample size of 22 participants required. Response scores for originality of initial response, originality, fluency, flexibility, and the total creativity score were analyzed using independent *t*-tests between the most- and least-creative groups.

### Visual search data analysis

The three most discriminating trials based on the greatest between-group differences in mean creativity scores were subjected to visual search analysis (cf. [12, 25]). An a priori task analysis in a form of a pilot study was conducted to try to identify the key discriminating period within each situation in order to better our understanding of the processes underpinning superior performance [26]. Based on the pilot analysis, it was determined that visual search data analysis begins when the play breaks forward and builds into a dangerous attacking scenario for each situation. Three main measures of visual search behavior were analyzed for this period:

#### Search rate

Three measures of search rate were examined including the mean fixation duration (in milliseconds), the mean number of fixations and the mean number of fixation locations per second. A fixation was defined as the participant’s point-of-gaze staying stationary on a particular location within a 1.5° of movement tolerance for three frames or more (>/ = 120 ms) [27]. Between-group differences across each of these three measures of search rate were analyzed separately using independent *t*-tests.

#### Percentage viewing time

The portion of time spent fixating on a particular area of interest on the display was calculated. The display was divided into seven fixation locations: *player in possession of the ball*; *ball* (i.e., ball flight); *space* (i.e., areas of free space on the pitch in which no player is located); *attacker*; *attacker in threatening position* (i.e., teammate in a dangerous position which could lead to a goal scoring opportunity if he received a pass); *defender*; and *other* category for visual saccades and fixations that did not match with the aforementioned areas. Percentage viewing time data were analyzed using a factorial two-way ANOVA with Group (most-, least-creative) as the between-participant factor and Fixation Location (player in possession of the ball, ball, space, attacker, attacker in a threatening position, defender, other) as within-participant factors. The Greenhouse-Geisser correction was employed in the case of violations of Mauchly’s test of sphericity. Effect sizes are reported using partial eta squared (*η*_*p*_^*2*^) in all instances and Cohen’s *d* for comparisons between two means. Post-hoc pairwise comparisons were conducted using the Bonferroni correction procedure in order to lower the significance threshold and avoid Type I errors [28].

#### Attacker in threatening position fixation

Following an exploratory analysis of the visual search data, a novel measure was identified. It referred to the moment of the first fixation on attackers in a threatening position during the attacking play. Between-group differences across moment of first fixation on attackers in a threatening position were analyzed separately using independent *t*-tests.

The alpha level (*p*) for statistical significance was set at .05 for all tests.

## Results

### Outcome data

The most-creative group recorded a significantly higher overall creative score on the soccer-specific tactical creativity test compared with the least creative, *t*(20) = 12.75, *p* < .001, *d* = 5.36. Also, the most-creative players produced more original decisions for the initial response, *t*(20) = 4.92, *p* < .001, *d* = 2.12, and for the responses given when the last frame was shown, *t*(20) = 3.93, *p* = .001, *d* = 1.68, as well as more appropriate, *t*(20) = 7.83, *p* < .001, *d* = 3.34, and flexible, *t*(20) = 8.01, *p* < .001, *d* = 3.40, tactical solutions. These data are presented in Table 1.

**Table 1:** Mean (SD) response scores for the soccer-specific tactical creativity test across groups.

	Group	
Measure	Most creative	Least creative
Originality (initial response)	3.30 (0.34)	2.65 (0.27)
Originality	2.78 (0.18)	2.47 (0.19)
Fluency	3.05 (0.23)	2.23 (0.26)
Flexibility	2.92 (0.34)	1.92 (0.24)
**Creativity score** (total, z-value)	**0.98 (0.34)**	**-0.87 (0.35)**

### Visual search data

#### Search rate

The descriptive statistics for search rate variables are presented in Table 2. There were significant group-based differences in the mean fixation duration, *t*(20) = -2.90, *p* = .009, *d* = 1.24, mean number of fixations, *t*(20) = 3.13, *p* = .005, *d* = 1.32, and the mean number of fixation locations per second, *t*(20) = 2.15, *p* = .044, *d* = 0.91. The visual search strategy of most-creative players involved more fixations of shorter duration to significantly more locations in the visual display when compared with the least-creative players.

**Table 2:** Mean (SD) fixation duration and number of fixations and fixation locations (per second) across groups.

	Group	
Search rate	Most creative	Least creative
Fixation duration (ms)	340 (72)	454 (109)
No. of fixations/s	2.71 (0.53)	2.08 (0.42)
No. of fixation locations/s	1.12 (0.15)	0.97 (0.17)

#### Percentage viewing time

**Fig 1:**
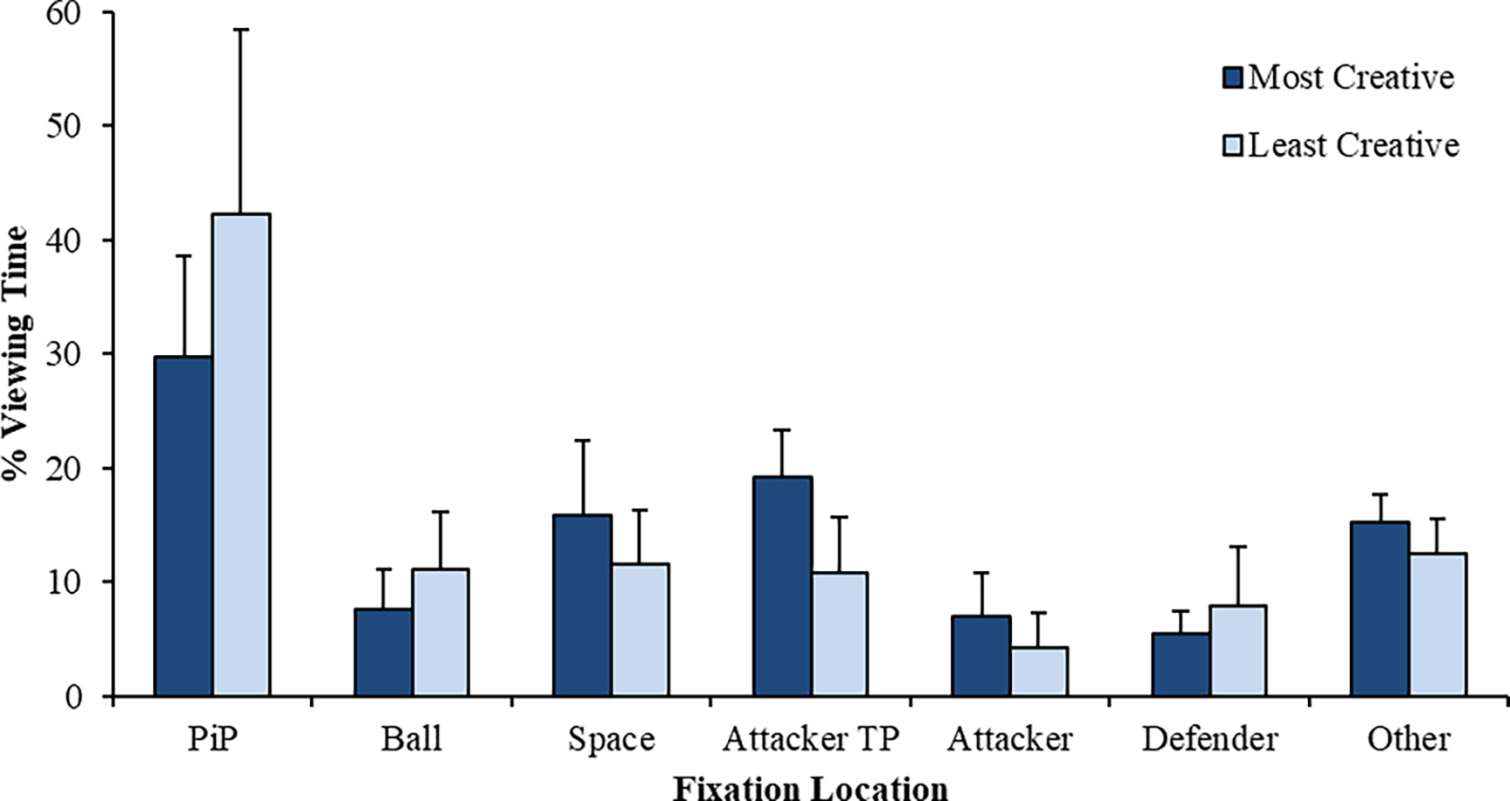
Mean (SD) percentage of time spent viewing each fixation location across groups (*PiP*, player in possession of the ball; *Attacker TP*, attacker in a threatening position).

The mean data for percentage viewing time are presented in Fig 1. There was a significant main effect for fixation location, *F*(1.78, 35.62) = 49.84, *p* < .001, η_p_^2^ = .71. Bonferroni-corrected pairwise comparisons demonstrated that participants spent significantly more time fixating the player in possession of the ball (*M* = 36.0%, *SD* = 14.2) compared with any other fixation location. This was followed by fixations on attackers in a threatening position (*M* = 14.9%, *SD* = 6.2), areas of free space (*M* = 13.6%, *SD* = 6.0), and other unclassified locations/visual saccades (*M* = 13.8%, *SD* = 3.1). No differences were evident between fixations on the ball (*M* = 9.3%, *SD* = 4.6), defenders (*M* = 6.7%, *SD* = 4.1), and other attacking team players (*M* = 5.6%, *SD* = 3.6).

A significant Group x Fixation Location interaction was observed, *F*(1.78, 35.62) = 5.47, *p* = .011, η_p_^2^ = .22. *Post-hoc* tests revealed that most-creative participants spent significantly more time fixating attackers in a threatening position compared with the least-creative participants (*M* = 19.1%, *SD* = 4.2 *vs*. *M* = 10.7%, *SD* = 4.9), *t*(20) = 4.27, *p* < .001, *d* = 1.84.

#### Attacker in threatening position fixation

**Fig 2:**
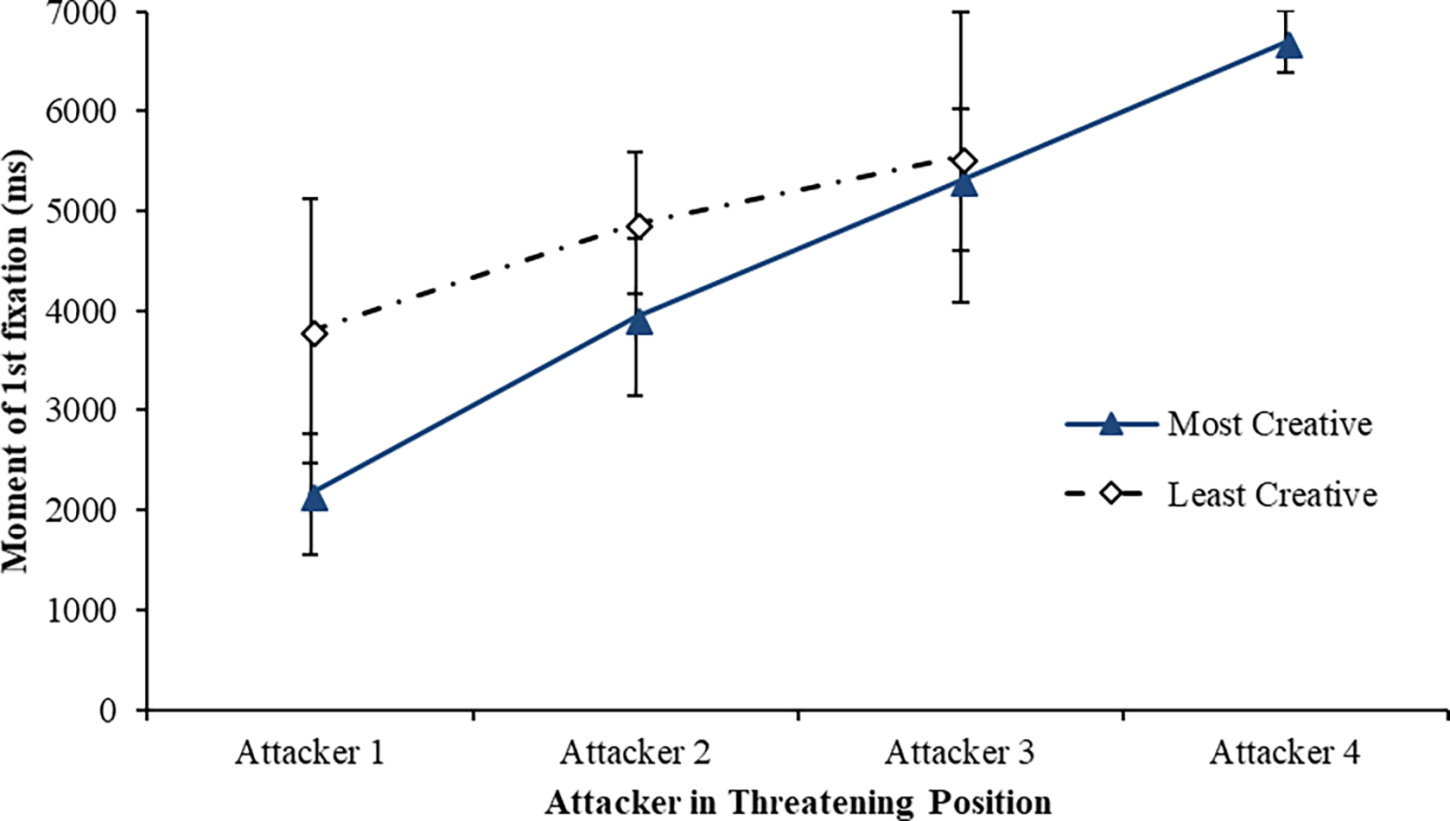
Mean (SD) moment of 1^st^ fixation in the attack on attackers in threatening position across groups.

The mean data for attacker in threatening position fixations are presented in Fig 2. There were significant group-based differences for the moment of first fixation on attackers in threatening position across groups. The most-creative players identified a first (*M* = 2,164 ms, *SD* = 606 *vs*. *M* = 3,798 ms, *SD* = 1,327), *t*(20) = -3.72, *p* = .001, *d* = 1.58, and a second attacker in a threatening position (*M* = 3,930 ms, *SD* = 789 *vs*. *M* = 4,872 ms, *SD* = 710), *t*(20) = -2.94, *p* = .008, *d* = 1.26, earlier on in the attacking play when compared with the least-creative group. Moreover, the most-creative players identified on average four attackers in threatening positions per trial as compared to only three attackers for the least-creative group.

## Discussion

We used a representative soccer video-based temporal occlusion creativity test to examine the visual search behaviors employed by skilled soccer players during open-play attacking situations offering a variety of possible solutions for the player in possession of the ball. Creativity performance scores were used to create two groups: most- and least-creative players. Visual search behaviors were collected to measure the perceptual processes underpinning superior creative performance on the task. First, we expected that the most-creative group would employ a search pattern involving more fixations of shorter duration and towards more informative locations of the display compared with least-creative players, indicating a broader breadth of attention. Second, we predicted that the most-creative players’ wider attentional focus would allow them to more effectively extract vital information cues earlier on in the play (e.g., teammates moving into dangerous positions), facilitating creative performance on the task.

In line with our hypotheses, the results showed that creativity-based between-group differences in decision making were underpinned by differences in visual search behaviors. We showed that most-creative players employed a different search strategy comprising of a greater number of fixations of shorter duration and directed towards more locations on the display. These findings were similar to those presented in previous work using eye-movement recording in soccer-specific convergent tactical thinking tasks (e.g., [13–16]), providing some evidence for the notion that creativity generally does require a certain level of domain-specific expertise [29]. Our results support the suggestion that most-creative players employ a broader attention focus taking in more relevant stimuli from a situation, which has been shown to facilitate the emergence of skilled creative behavior [10, 30]. According to Friedman, Fishbach, Förster, and Werth [31], a narrow focus of attention limits the amount of stimuli and information that can be acquired and processed, leading to players missing important game-relevant information; whereas, a wide breath of attention makes it possible to associate different stimuli that may initially appear to be irrelevant [9].

Furthermore, the visual search data showed the timing of fixating on key information as the attacking play unfolded differed between groups, specifically the moment of first fixation on other key attackers in or moving into a threatening position. Results showed that most-creative players could not only detect a greater number of teammates in positions that might lead to a goal scoring opportunity if they received the ball, but they also did so significantly earlier in the attacking situations when compared with the least-creative counterparts. The presented results provide some preliminary evidence that superior creative performance of most-creative players appears to be related to early perception of highly relevant cues. A broader attention focus appears to be necessary in order to perceive unexpected objects, such as teammates in dangerous positions, which could potentially initiate unique and original solutions [9, 10].

Findings have implications for practice and provide support for the benefit of designing practice environments that cause players to use a wide breadth of attention in order to promote the development of creative expertise (e.g., see [30]). While this research has uncovered some novel findings, it focused only on the underlying perceptual processes underpinning superior creativity on the task. In the future, researchers should also attempt to identify how performers translate the information perceived from the visual display into appropriate creative decisions, thus providing greater insight into the important cognitive processes that mediate and interlink perception and superior creative behavior [32].

In summary, creativity-based between-group differences were underpinned by quantitative differences in visual search strategy. Most-creative players employed a broader focus of attention that included a greater number of fixations of shorter duration and towards more informative areas of the display than their least-creative counterparts. The superior performance of the most-creative group was also supported by the earlier detection of key relevant cues, specifically attacking teammates in threatening positions. Findings provide an important contribution towards the development of more refined models of tactical creativity and expertise in sport.

## Supporting information

S1 Dataset Creativity performance scores. (SAV)

S2 Dataset Search rate. (SAV)

S3 Dataset Percentage viewing time. (SAV)

S4 Dataset Attacker in threatening position fixation. (SAV)
